# Supplementary material for: Mechanical frequency control in inductively coupled electromechanical systems
Source: Sci Rep. 2022 Jan 31;12:1608. doi: 10.1038/s41598-022-05438-x (PMC8803975; doi:10.1038/s41598-022-05438-x)
Supplement: Supplementary file 1 — Supplementary Information. [file 41598_2022_5438_MOESM1_ESM.pdf]

Supplemental Material: Mechanical frequency control in inductively coupled electromechanical systems

Thomas Luschmann,[1,2,3] Philip Schmidt,[1,2,4] Frank Deppe,[1,2,3] Achim Marx,[1] Alvaro Sanchez,[5] Rudolf Gross,[1,2,3] and Hans Huebl[1,2,3]

[1] *Walther-Meißner-Institut, Bayerische Akademie der Wissenschaften, Walther-Meißner-Str.8, 85748 Garching, Germany*

[2] *Physik-Department, Technische Universität München, James-Franck-Str.1, 85748 Garching, Germany*

[3] *Munich Center for Quantum Science and Technology, Schellingstr.4, 80799, Munich, Germany*

[4] *Present address: Institute for Quantum Optics and Quantum Information, Austrian Academy of Sciences, 1090 Vienna, Austria*

[5] *Department of Physics, Universitat Autonoma de Barcelona, 08193 Bellaterra, Catalonia, Spain*

(Dated: January 4, 2022)

Supplementary Note 1: Device parameters

Table T1 contains an overview of the relevant device parameters that were used in the fitting of measurement data to Eq. (2) of the main text. We note that the asymmetry has been assumed to account for the usual, small deviations occuring in Josephson junction fabrication.

Supplementary Note 2: Discussion of magnetic torque effects as possible origin of the additional frequency shift

As a possible explanation of the additional mechanical frequency shift observed in the experiment (see Fig. 3 and discussion in the main text), we considered the flux captured by the SQUID loop to act as a magnetic moment m_{SQ}. This could lead to effects within the device known from cantilever torque magnetometry [2–4]. The loop, which can be well approximated as a single square coil, hosts a magnetic moment m_{SQ} due the presence of captured flux. This m_{SQ} points along the out-of-plane direction of the chip [5]. Under the assumption of a slightly imperfect alignment of the magnetic field, the torque $\tau = m_{\mathrm{SQ}} \times B_{\mathrm{IP}}$ can be associated with a position dependent force on the string, which could be responsible for the shift in resonance frequency. However, the effect of this torque would be expected to depend on the number of flux quanta in the SQUID loop.

To this end we performed additional measurements probing the mechanical frequency of the system at different flux bias points, which correspond to a different number of flux quanta present in the SQUID loop. The data is presented in Fig. S1. The resulting $\Omega_0/2\pi = 5.800\,36\,\mathrm{MHz}$, extracted from fits to Eq. (2) of the main text, is identical for both datasets, suggesting that the observed frequency shift does not depend on the number of flux quanta in the SQUID loop, ruling out the magnetic torque hypothesis.

Parameter	Value	Reference / Comment
Shape factor λ	0.95	Ref. [1]
Asymmetry α	0.01	
Mass m_{r}	0.6 pg	The effective mass of the string
Length l	20 μm	

TABLE T1. Summary of relevant device parameters for the calculation of the expected frequency shift according to Eq. (2) of the main text.

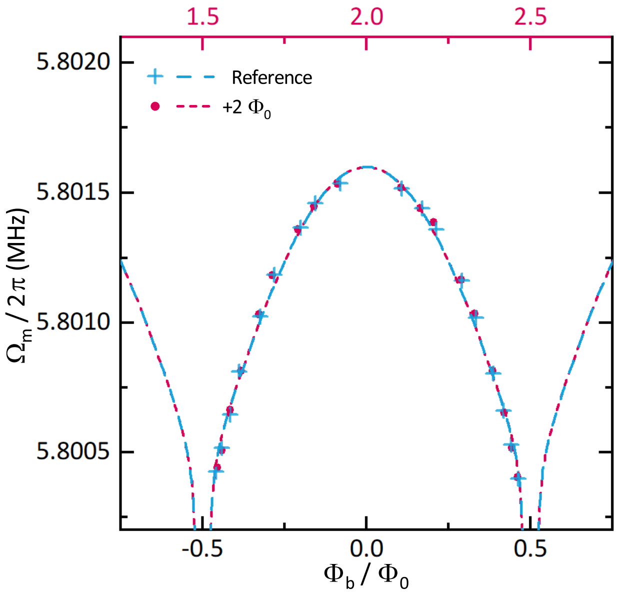

FIG. S1. Mechanical resonance frequencies at various values of the flux bias Φ_{b}, measured at a constant in-plane field $B_{\mathrm{IP}} = 35\,\mathrm{mT}$. After the reference measurement (blue crosses) Φ_{b} was increased until the microwave resonator's frequency experiences two complete tuning cycles, i.e. the SQUID loop should contain two additional flux quanta Φ_0. Subsequently, the measurement was repeated for the new bias points (red dots, upper x-axis). Both datasets were fitted independently according to Eq. (2) of the main text (dashed lines) and result in a best fit for $\Omega_0/2\pi = 5.800\,36\,\mathrm{MHz}$. Statistical error bars are smaller than the symbol size.

REFERENCES

[1] P. Schmidt, M. T. Amawi, S. Pogorzalek, F. Deppe, A. Marx, R. Gross, and H. Huebl, Commun. Phys. **3**, 233 (2020).
[2] A. Kamra, S. von Hoesslin, N. Roschewsky, J. Lotze, M. Schreier, R. Gross, S. T. Goennenwein, and H. Huebl, Eur. Phys. J. B **88**, 224 (2015).
[3] A. Kamra, M. Schreier, H. Huebl, and S. T. B. Goennenwein, Phys. Rev. B **89**, 184406 (2014).
[4] I. Petkovic, A. Lollo, and J. G. E. Harris, Phys. Rev. Lett. **125**, 067002 (2020).
[5] M. Misakian, J. Res. Natl. Inst. Stand. Technol. **105**, 557 (2000).
